# Synthesis and crystal structure of tri­ethyl­ammonium hexa­bromido­uranate(IV) di­chloro­methane monosolvate

**DOI:** 10.1107/S205698902001169X

**Published:** 2020-09-04

**Authors:** Holger Lars Deubner, Marcel Koester, Carsten von Haenisch, Florian Kraus

**Affiliations:** a Anorganische Chemie, Fachbereich Chemie, Philipps-Universität Marburg, Hans-Meerwein-Strasse 4, 35032 Marburg, Germany

**Keywords:** crystal structure, uranium, bromide, tri­ethyl­amine

## Abstract

Tri­ethyl­ammonium hexa­bromido­uranate(IV) di­chloro­methane monosolvate, [(C_2_H_5_)_3_NH]_2_[UBr_6_]·CH_2_Cl_2_, was obtained in the form of dark-brown crystals from the reaction of uranium penta­bromide with NEt_3_ and ethyl­ene glycol in di­chloro­methane at low temperature. During the progress of the reaction, the reduction of uranium(V) to uranium(IV) was observed, whose associated oxidation product could not be identified. The uranium atom of the [UBr_6_]^2–^ anion is coordinated by six bromido ligands in the shape of an octa­hedron. Between cations, anion and solvent mol­ecules of crystallization, numerous C—H⋯Hal hydrogen-bond-like inter­actions are present, leading to a three-dimensional network structure.

## Abstract 2

The synthesis and crystal structure determination of (Et_3_NH)_2_[UBr_6_]·CH_2_Cl_2_ is reported.

## Chemical context   

Starting in the 1950s, a variety of hexa­chlorido­uranates(IV) with organic cations have been investigated and described [CSD Database, Version 2.0.4, accessed 19.05.2020 (Groom *et al.*, 2016 ▸); Staritzky & Singer, 1952 ▸). Examples of this type of compound are (Me_4_N)_2_[UCl_6_], (Ph_4_As)_2_[UCl_6_]·CH_2_Cl_2_, or the 4,4′-bipyridin-1-ium hexa­chlorido­uranate(IV) (C_10_H_10_N_2_)[UCl_6_] (Autillo & Wilson, 2017 ▸; Müller *et al.*, 1984 ▸; Wacker *et al.*, 2019 ▸). All these compounds have a slightly distorted octa­hedron-shaped [UCl_6_]^2–^ coordination polyhedron in common with Cl—U—Cl angles close to 90° and some of them, like [BuMeIm]_2_[UCl_6_], feature hydrogen-bonding networks or weak hydrogen inter­actions (Nikitenko *et al.*, 2007 ▸). Examples of hexa­bromido­uranates(IV) with organic cations are (PPh_4_)_2_[UBr_6_]·4CH_3_CN (Bohrer *et al.*, 1988 ▸), [P(C_6_H_5_)_3_C_2_H_5_]_2_[UBr_6_] (Caira *et al.*, 1978 ▸) or (Ph_3_EtP)_2_[UBr_6_] (Caira *et al.*, 1978 ▸). As in the case of the hexa­chlorido­uranates(IV), the [UBr_6_]^2–^ octa­hedra show a slight distortion and the compounds feature an extended network of hydrogen bonds. The only two structurally elucidated examples of hexa­bromido­uranate(V) anions, [UBr_6_]^−^, that show significantly shorter U—Br distances compared to [UBr_6_]^2–^ anions are (Ph_4_P)[UBr_6_] and (Ph_4_P)[UBr_6_]·2CCl_4_ (Bohrer *et al.*, 1988 ▸). These two compounds as well as (PPh_4_)[UBr_6_]·CH_2_Cl_2_ serve as examples for the stability of uranium(V) in organic solvents; however, a reduction of U^V^ to U^IV^ was observed upon removal of the solvent (Bohrer *et al.*, 1988 ▸). During this reaction, elemental bromine was formed *via* oxidation besides the adduct UBr_4_·CH_3_CN (Bohrer *et al.*, 1988 ▸).

We, however, observe reduction of UBr_5_ to uranium(IV) as [UBr_6_]^2–^ and the protonation of NEt_3_. It is plausible that ethyl­ene glycol serves as the proton source for the formation of the HNEt_3_^+^ cations; however, we do not know where the glycolate anions end up. We also do not know what the reducing agent for the reduction of U^V^ to U^IV^) is, or if UBr_5_ is simply unstable under these conditions and is converted to UBr_4_ and 0.5 Br_2_. We also do not know how UBr_5_ is dissolved, that is, whether U_2_Br_10_ mol­ecules or other mono- or polynuclear complexes, such as of gylcolates, are present in solution. Elemental bromine may be present within the brown solution and act as an oxidizing agent under the formation of the Br^−^ anions required to constitute the [UBr_6_]^2–^ anions. For the reactions to be stoichiometric, some leftover U species should have been formed that we did not observe. In summary, the detailed formation of the title compound (Et_3_NH)_2_[UBr_6_]·CH_2_Cl_2_ remains unclear.
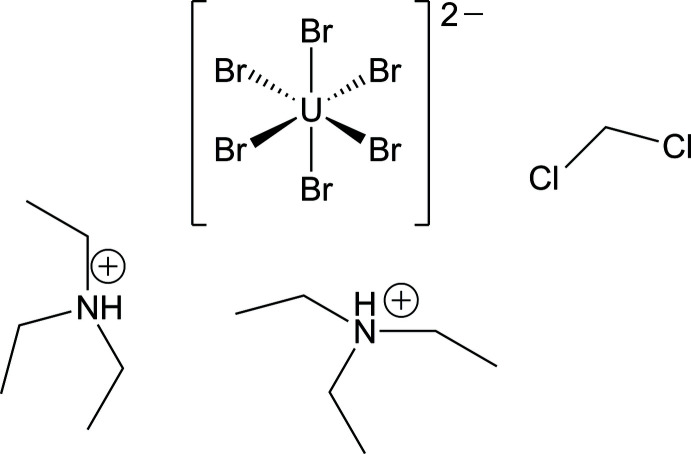


## Structural commentary   

**Figure 1:**
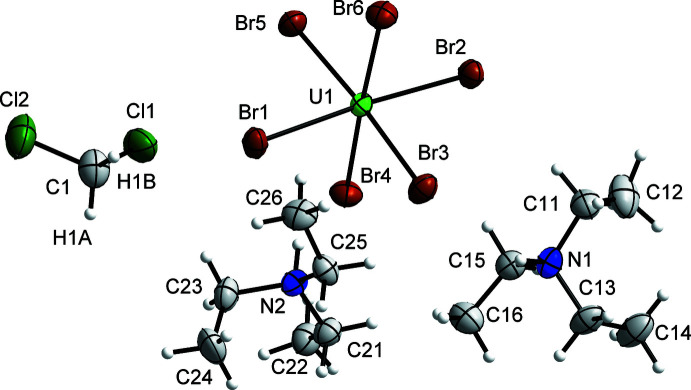
Section of the crystal structure of (Et_3_NH)_2_[UBr_6_]·CH_2_Cl_2_, illustrating the asymmetric unit. Displacement ellipsoids are shown at the 70% probability level at 100 K and H atoms are drawn with an arbitrary radius.

The compound tri­ethyl­ammonium hexa­bromido­uranate(IV)–di­chloro­methane (1/1) (Et_3_NH)_2_[UBr_6_]·CH_2_Cl_2_ crystallizes in the monoclinic crystal system, space group *P*2_1_/*n* (No. 14), with the lattice parameters *a* = 10.7313 (3), *b* = 17.4534 (4), *c* = 15.0090 (5) Å, *β* = 92.0550 (10)°, *V* = 2809.34 (14) Å^3^, *Z* = 4 at *T* = 100 K. The uranium atom of the cation is coordinated by six bromine ligands in the shape of a slightly distorted octa­hedron. The atomic distances between the uranium atom and the bromido ligands range from 2.7562 (4) to 2.7847 (5) Å (Table 1 ▸). The Br—U—Br angles in the octa­hedron-like polyhedron range from 86.519 (13) to 94.879 (14) and show a quite significant distortion from the ideal angle of 90°. These atomic distances and angles are in good agreement with the compounds reported previously (Bohrer *et al.*, 1988 ▸; Caira *et al.*, 1978 ▸). The atomic distances of the two symmetry-independent tri­ethyl­ammonium cations are in good agreement with each other, as well as with the literature, for example in bis­(tri­ethyl­ammonium) tetra­chlorido­dioxidouranium(VI) (Gatto *et al.*, 2003 ▸; Serezhkina *et al.*, 2010 ▸; Bois *et al.*, 1976 ▸). Fig. 1 ▸ shows a section of the crystal structure.

## Supra­molecular features   

**Figure 2:**
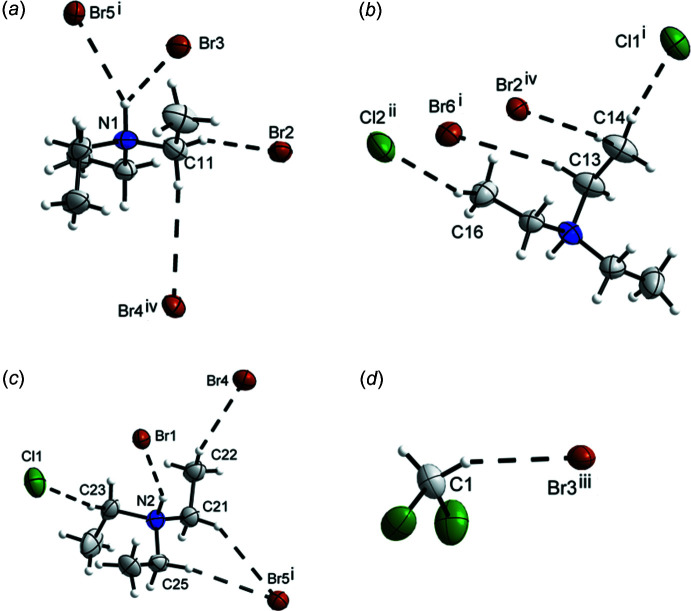
The hydrogen bonds and hydrogen-bond-like inter­actions (dashed lines) present in the structure of the title compound. (*a*) and (*b*) show the inter­actions of the HNEt^+^ cation with N1, (*c*) of the HNEt^+^ cation with N2, and (*d*) shows the inter­actions of DCM. Displacement ellipsoids within each subfigure are shown at the 70% probability level at 100 K and H atoms are drawn with an arbitrary radius. See Table 2 ▸ for symmetry operators.

Sections of the crystal structure, illustrating the hydrogen-bonding situation, are shown in Fig. 2 ▸. The hydrogen bonds were inspected visually and those with angles less than 134° were removed from the analysis. The Br3 and Br5 atoms of the [UBr_6_]^2–^ anion act as acceptors for the bifurcated N—H⋯Br hydrogen bond. The other HNEt_3_^+^ cation (with N2) also forms a N—H⋯Br hydrogen bond, however, not bifurcated. Hydrogen-bond lengths and angles are given in Table 2 ▸. Furthermore, C—H⋯Hal hydrogen-bond-like inter­actions between the HNEt_3_^+^ cations and the Br atoms of the [UBr_6_]^2–^ anion as well as to the Cl atoms of the di­chloro­methane mol­ecules are also present. Overall, a three-dimensional hydrogen-bonded network results. An overview of the hydrogen-bond lengths between the cations, anion and solvent mol­ecule in the compound reported here is given in Table 2 ▸. The C–H⋯Br hydrogen bonds in (Ph_3_EtP)_2_[UBr_6_] (Caira *et al.*, 1978 ▸) range from 2.782 (1) to 3.504 (2) Å. An example for N–H⋯Br hydrogen bonds is (C_6_H_8_NS_3_)_2_[UBr_6_] (Conradi *et al.*, 1986 ▸), with lengths of 2.81 (9) Å for the inter­actions. These bond lengths are comparable with the presented data.

## Synthesis and crystallization   

50 mg of UBr_5_ (0.08 mmol, 1.00 eq) were dissolved in 2 mL of predried DCM and 0.06 mL of NEt_3_ (40 mg, 0.39 mmol, 5.00 eq.) were added. Then, after stirring briefly, 0.01 mL of ethyl­ene glycol (10 mg, 0.20 mmol, 2.50 eq.) were added dropwise. After two h, the reaction mixture was filtered and the obtained brown filtrate was cooled to 241 K. The product was obtained in crystalline form after three days as brown plates. A selected crystal was investigated by X-ray diffraction. As only a few crystals precipitated from the cold filtrate, the yield could not be determined, but it can be assumed that it was rather low. No further analysis was carried out on the few minute crystals or the filtrate. UBr_5_ was synthesized according to the literature (Deubner *et al.*, 2019 ▸).

## Refinement   

Crystal data, data collection and structure refinement details are summarized in Table 3 ▸. Hydrogen atoms were positioned geometrically (N—H = 1.00Å, C—H = 0.98–0.99Å) refined using a riding model with *U*_iso_(H) = 1.2*U*_eq_(N,C) or 1.5*U*_eq_(C_meth­yl_). The maximum and minimum residual electron densities are located close to the U atom at distances of 0.77 and 1.19 Å, respectively.

## Supplementary Material

Crystal structure: contains datablock(s) I. DOI: 10.1107/S205698902001169X/zl2792sup1.cif

CCDC reference: 2025374

Additional supporting information:  crystallographic information; 3D view; checkCIF report

## Figures and Tables

**Table 1:** Selected inter­atomic distances *d* (Å) for (Et_3_NH)_2_[UBr_6_]

Atom 1	Atom 2	*d*
U1	Br1	2.7835 (4)
	Br2	2.7562 (4)
	Br3	2.7847 (5)
	Br4	2.7613 (4)
	Br5	2.7631 (5)
	Br6	2.7658 (4)
N1	C11	1.500 (6)
	C13	1.514 (6)
	C15	1.515 (5)
N2	C21	1.508 (5)
	C23	1.517 (6)
	C25	1.504 (5)
C1	Cl1	1.756 (5)
	Cl2	1.751 (5)
C11	C12	1.521 (7)
C13	C14	1.508 (6)
C15	C16	1.506 (6)
C21	C22	1.509 (6)
C23	C24	1.511 (7)
C25	C26	1.503 (6)

**Table 2:** Hydrogen-bond geometry (Å, °)

*D*—H⋯*A*	*D*—H	H⋯*A*	*D*⋯*A*	*D*—H⋯*A*
N1—H1⋯Br3	1.00	2.71	3.480 (3)	134
N1—H1⋯Br5^i^	1.00	2.83	3.658 (3)	141
C11—H11*A*⋯Br2	0.99	3.13	4.070 (5)	158
C11—H11*B*⋯Br4^ii^	0.99	3.24	4.184 (4)	160
C13—H13*B*⋯Br6^i^	0.99	3.38	4.339 (5)	163
C14—H14*A*⋯Br2^ii^	0.98	3.38	4.350 (6)	173
C14—H14*B*⋯Cl1^i^	0.98	2.95	3.922 (5)	169
C16—H16*B*⋯Cl2^iii^	0.98	2.97	3.904 (5)	160
N2—H2⋯Br1	1.00	2.59	3.499 (4)	152
C21—H21*B*⋯Br5^i^	0.99	3.12	4.005 (5)	150
C22—H22*C*⋯Br4	0.98	3.20	4.139 (5)	162
C23—H23*A*⋯Cl1	0.99	2.88	3.865 (5)	171
C25—H25*B*⋯Br5^i^	0.99	3.00	3.886 (4)	149
C1—H1*B*⋯Br3^iv^	0.99	2.81	3.729 (5)	155

Symmetry codes: (i) 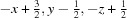; (ii) 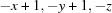; (iii) 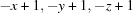; (iv) 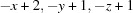.

**Table 3:** Experimental details

Crystal data	
Chemical formula	(C_6_H_16_N)_2_[UBr_6_]·CH_2_Cl_2_
*M*_r_	1006.81
Crystal system, space group	Monoclinic, *P*2_1_/*n*
Temperature (K)	100
*a*, *b*, *c* (Å)	10.7318 (4), 17.4541 (4), 15.0082 (4)
β (°)	92.055 (1)
*V* (Å^3^)	2809.44 (14)
*Z*	4
Radiation type	Mo *K*α
μ (mm^−1^)	14.50
Crystal size (mm)	0.1 × 0.1 × 0.05

Data collection	
Diffractometer	Stoe IPDS 2T
Absorption correction	Numerical (*X-RED32*; Stoe & Cie, 2009 ▸)
*T*_min_, *T*_max_	0.049, 0.527
No. of measured, independent and observed [*I* > 2σ(*I*)] reflections	35494, 5945, 5307
*R*_int_	0.065
(sin θ/λ)_max_ (Å^−1^)	0.634

Refinement	
*R*[*F* ^2^ > 2σ(*F* ^2^)], *wR*(*F* ^2^), *S*	0.025, 0.059, 1.04
No. of reflections	5945
No. of parameters	223
H-atom treatment	H-atom parameters constrained
Δρ_max_, Δρ_min_ (e Å^−3^)	1.52, −1.12

Computer programs: *X-AREA* (Stoe & Cie, 2018 ▸), *SHELXT2018/3* (Sheldrick, 2015*a* ▸), *SHELXL2018/3* (Sheldrick, 2015*b* ▸) and *DIAMOND* (Brandenburg, 2019 ▸).

## supplementary crystallographic information

### Crystal data

**Table:**

(C_6_H_16_N)_2_[UBr_6_]·CH_2_Cl_2_	*F*(000) = 1848
*M**_r_* = 1006.81	*D*_x_ = 2.380 Mg m^−^^3^
Monoclinic, *P*2_1_/*n*	Mo *K*α radiation, λ = 0.71073 Å
*a* = 10.7318 (4) Å	Cell parameters from 72381 reflections
*b* = 17.4541 (4) Å	θ = 5.1–53.6°
*c* = 15.0082 (4) Å	µ = 14.50 mm^−^^1^
β = 92.055 (1)°	*T* = 100 K
*V* = 2809.44 (14) Å^3^	Plate, dark brown
*Z* = 4	0.1 × 0.1 × 0.05 mm

### Data collection

**Table:**

STOE IPDS 2T diffractometer	5945 independent reflections
Radiation source: sealed X-ray tube, 12 x 0.4 mm long-fine focus	5307 reflections with *I* > 2σ(*I*)
Planar graphite monochromator	*R*_int_ = 0.065
Detector resolution: 6.67 pixels mm^-1^	θ_max_ = 26.8°, θ_min_ = 2.6°
rotation method, ω scans	*h* = −13→13
Absorption correction: numerical (X-Red32; Stoe & Cie, 2009)	*k* = −22→22
*T*_min_ = 0.049, *T*_max_ = 0.527	*l* = −19→18
35494 measured reflections	

### Refinement

**Table:**

Refinement on *F*^2^	Primary atom site location: dual
Least-squares matrix: full	Secondary atom site location: difference Fourier map
*R*[*F*^2^ > 2σ(*F*^2^)] = 0.025	Hydrogen site location: inferred from neighbouring sites
*wR*(*F*^2^) = 0.059	H-atom parameters constrained
*S* = 1.04	*w* = 1/[σ^2^(*F*_o_^2^) + (0.0252*P*)^2^ + 6.7371*P*] where *P* = (*F*_o_^2^ + 2*F*_c_^2^)/3
5945 reflections	(Δ/σ)_max_ = 0.002
223 parameters	Δρ_max_ = 1.52 e Å^−^^3^
0 restraints	Δρ_min_ = −1.12 e Å^−^^3^

### Special details

**Table:**

Geometry. All esds (except the esd in the dihedral angle between two l.s. planes) are estimated using the full covariance matrix. The cell esds are taken into account individually in the estimation of esds in distances, angles and torsion angles; correlations between esds in cell parameters are only used when they are defined by crystal symmetry. An approximate (isotropic) treatment of cell esds is used for estimating esds involving l.s. planes.

### Fractional atomic coordinates and isotropic or equivalent isotropic displacement parameters (Å^2^)

**Table:**

	*x*	*y*	*z*	*U*_iso_*/*U*_eq_	
U1	0.70763 (2)	0.60417 (2)	0.25294 (2)	0.01693 (5)	
Br1	0.69695 (4)	0.57164 (3)	0.43407 (3)	0.02583 (10)	
Br2	0.71934 (4)	0.62538 (3)	0.07145 (3)	0.02426 (10)	
Br3	0.79682 (4)	0.45554 (3)	0.23313 (3)	0.02729 (10)	
Br4	0.46676 (4)	0.55130 (3)	0.22569 (3)	0.02739 (10)	
Br5	0.61892 (4)	0.74956 (2)	0.28651 (3)	0.02490 (10)	
Br6	0.95008 (4)	0.65689 (3)	0.27283 (3)	0.02697 (10)	
Cl1	0.80839 (13)	0.55597 (8)	0.65867 (8)	0.0411 (3)	
Cl2	0.83159 (13)	0.58050 (10)	0.85069 (9)	0.0513 (4)	
N1	0.7139 (3)	0.3577 (2)	0.0379 (2)	0.0233 (8)	
H1	0.755481	0.354489	0.098517	0.028*	
N2	0.7104 (3)	0.3716 (2)	0.4459 (2)	0.0232 (8)	
H2	0.705708	0.424063	0.419282	0.028*	
C1	0.8582 (4)	0.5179 (3)	0.7622 (3)	0.0350 (11)	
H1A	0.813690	0.469171	0.772342	0.042*	
H1B	0.948470	0.506395	0.761006	0.042*	
C11	0.7928 (4)	0.4099 (3)	−0.0159 (3)	0.0305 (10)	
H11A	0.798906	0.460352	0.014011	0.037*	
H11B	0.751630	0.417679	−0.075281	0.037*	
C12	0.9236 (4)	0.3788 (4)	−0.0280 (4)	0.0439 (14)	
H12A	0.960175	0.363457	0.030062	0.066*	
H12B	0.975348	0.418558	−0.054084	0.066*	
H12C	0.919360	0.334222	−0.067749	0.066*	
C13	0.7052 (5)	0.2763 (3)	0.0032 (3)	0.0306 (10)	
H13A	0.789983	0.253987	0.003254	0.037*	
H13B	0.655161	0.245525	0.044208	0.037*	
C14	0.6472 (5)	0.2707 (3)	−0.0896 (3)	0.0387 (12)	
H14A	0.561944	0.290737	−0.089751	0.058*	
H14B	0.645537	0.216946	−0.108520	0.058*	
H14C	0.696590	0.300684	−0.130777	0.058*	
C15	0.5882 (4)	0.3948 (3)	0.0504 (3)	0.0244 (9)	
H15A	0.601033	0.447669	0.072758	0.029*	
H15B	0.542776	0.397995	−0.008087	0.029*	
C16	0.5098 (4)	0.3514 (3)	0.1146 (3)	0.0322 (11)	
H16A	0.484504	0.302216	0.087991	0.048*	
H16B	0.435415	0.381437	0.127367	0.048*	
H16C	0.558378	0.342157	0.170045	0.048*	
C21	0.6301 (4)	0.3208 (3)	0.3864 (3)	0.0260 (9)	
H21A	0.626464	0.269114	0.413275	0.031*	
H21B	0.669358	0.315813	0.327985	0.031*	
C22	0.4989 (4)	0.3505 (3)	0.3715 (3)	0.0310 (10)	
H22A	0.455318	0.348800	0.427708	0.046*	
H22B	0.454521	0.318541	0.327033	0.046*	
H22C	0.501713	0.403488	0.349993	0.046*	
C23	0.6641 (4)	0.3789 (3)	0.5399 (3)	0.0296 (10)	
H23A	0.709967	0.420914	0.570805	0.036*	
H23B	0.574683	0.392884	0.536600	0.036*	
C24	0.6800 (6)	0.3064 (3)	0.5941 (3)	0.0445 (13)	
H24A	0.636358	0.264126	0.563271	0.067*	
H24B	0.644987	0.313862	0.652859	0.067*	
H24C	0.768859	0.294031	0.601240	0.067*	
C25	0.8449 (4)	0.3475 (3)	0.4436 (3)	0.0248 (9)	
H25A	0.854298	0.296273	0.471340	0.030*	
H25B	0.868916	0.343198	0.380740	0.030*	
C26	0.9315 (4)	0.4027 (3)	0.4915 (3)	0.0325 (11)	
H26A	0.918147	0.454356	0.467310	0.049*	
H26B	1.018010	0.387174	0.483028	0.049*	
H26C	0.914733	0.402615	0.555219	0.049*	

### Atomic displacement parameters (Å^2^)

**Table:**

	*U*^11^	*U*^22^	*U*^33^	*U*^12^	*U*^13^	*U*^23^
U1	0.01705 (8)	0.01736 (9)	0.01627 (7)	0.00032 (6)	−0.00099 (5)	−0.00022 (5)
Br1	0.0311 (2)	0.0274 (2)	0.01893 (18)	0.00404 (18)	0.00139 (16)	0.00121 (17)
Br2	0.0261 (2)	0.0274 (2)	0.01914 (18)	−0.00121 (17)	−0.00094 (16)	−0.00011 (16)
Br3	0.0308 (2)	0.0229 (2)	0.0278 (2)	0.00371 (17)	−0.00318 (17)	−0.00230 (17)
Br4	0.02038 (19)	0.0287 (2)	0.0329 (2)	−0.00374 (17)	−0.00104 (17)	0.00114 (18)
Br5	0.0270 (2)	0.0205 (2)	0.0272 (2)	0.00144 (17)	0.00110 (16)	−0.00114 (17)
Br6	0.02052 (19)	0.0305 (2)	0.0297 (2)	−0.00311 (17)	−0.00316 (16)	−0.00108 (18)
Cl1	0.0534 (7)	0.0411 (7)	0.0285 (6)	−0.0082 (6)	−0.0010 (5)	0.0058 (5)
Cl2	0.0456 (7)	0.0706 (10)	0.0377 (7)	−0.0045 (7)	0.0036 (6)	−0.0211 (7)
N1	0.0237 (17)	0.026 (2)	0.0199 (17)	0.0037 (15)	−0.0050 (14)	−0.0013 (15)
N2	0.0231 (17)	0.0223 (19)	0.0242 (18)	−0.0015 (15)	−0.0009 (14)	−0.0026 (15)
C1	0.027 (2)	0.047 (3)	0.031 (2)	0.004 (2)	−0.0007 (19)	−0.003 (2)
C11	0.027 (2)	0.036 (3)	0.028 (2)	−0.005 (2)	−0.0007 (18)	−0.002 (2)
C12	0.027 (2)	0.069 (4)	0.036 (3)	−0.001 (3)	0.006 (2)	−0.007 (3)
C13	0.038 (2)	0.020 (2)	0.033 (2)	0.0048 (19)	−0.008 (2)	0.0000 (19)
C14	0.053 (3)	0.029 (3)	0.033 (2)	0.004 (2)	−0.013 (2)	−0.005 (2)
C15	0.021 (2)	0.025 (2)	0.027 (2)	0.0018 (17)	−0.0027 (17)	−0.0005 (18)
C16	0.026 (2)	0.040 (3)	0.031 (2)	−0.003 (2)	−0.0024 (18)	0.005 (2)
C21	0.024 (2)	0.027 (2)	0.028 (2)	−0.0003 (18)	−0.0003 (17)	−0.0036 (18)
C22	0.024 (2)	0.035 (3)	0.034 (2)	0.001 (2)	0.0015 (18)	−0.006 (2)
C23	0.025 (2)	0.037 (3)	0.027 (2)	0.003 (2)	0.0051 (18)	−0.009 (2)
C24	0.058 (3)	0.045 (3)	0.032 (3)	−0.001 (3)	0.021 (2)	0.006 (2)
C25	0.0187 (19)	0.030 (3)	0.026 (2)	0.0036 (18)	0.0041 (16)	0.0029 (18)
C26	0.024 (2)	0.038 (3)	0.035 (2)	−0.004 (2)	−0.0069 (19)	0.000 (2)

### Geometric parameters (Å, º)

**Table:**

U1—Br2	2.7562 (4)	C14—H14A	0.9800
U1—Br4	2.7613 (4)	C14—H14B	0.9800
U1—Br5	2.7631 (5)	C14—H14C	0.9800
U1—Br6	2.7658 (4)	C15—C16	1.506 (6)
U1—Br1	2.7835 (4)	C15—H15A	0.9900
U1—Br3	2.7847 (5)	C15—H15B	0.9900
Cl1—C1	1.756 (5)	C16—H16A	0.9800
Cl2—C1	1.751 (5)	C16—H16B	0.9800
N1—C11	1.500 (6)	C16—H16C	0.9800
N1—C13	1.514 (6)	C21—C22	1.509 (6)
N1—C15	1.515 (5)	C21—H21A	0.9900
N1—H1	1.0000	C21—H21B	0.9900
N2—C25	1.504 (5)	C22—H22A	0.9800
N2—C21	1.508 (5)	C22—H22B	0.9800
N2—C23	1.517 (6)	C22—H22C	0.9800
N2—H2	1.0000	C23—C24	1.511 (7)
C1—H1A	0.9900	C23—H23A	0.9900
C1—H1B	0.9900	C23—H23B	0.9900
C11—C12	1.521 (7)	C24—H24A	0.9800
C11—H11A	0.9900	C24—H24B	0.9800
C11—H11B	0.9900	C24—H24C	0.9800
C12—H12A	0.9800	C25—C26	1.503 (6)
C12—H12B	0.9800	C25—H25A	0.9900
C12—H12C	0.9800	C25—H25B	0.9900
C13—C14	1.508 (6)	C26—H26A	0.9800
C13—H13A	0.9900	C26—H26B	0.9800
C13—H13B	0.9900	C26—H26C	0.9800
			
Br2—U1—Br4	88.519 (13)	H14A—C14—H14B	109.5
Br2—U1—Br5	94.879 (14)	C13—C14—H14C	109.5
Br4—U1—Br5	90.407 (14)	H14A—C14—H14C	109.5
Br2—U1—Br6	89.194 (13)	H14B—C14—H14C	109.5
Br4—U1—Br6	177.674 (14)	C16—C15—N1	112.6 (4)
Br5—U1—Br6	90.214 (14)	C16—C15—H15A	109.1
Br2—U1—Br1	175.943 (14)	N1—C15—H15A	109.1
Br4—U1—Br1	90.307 (14)	C16—C15—H15B	109.1
Br5—U1—Br1	89.011 (13)	N1—C15—H15B	109.1
Br6—U1—Br1	91.945 (13)	H15A—C15—H15B	107.8
Br2—U1—Br3	89.519 (13)	C15—C16—H16A	109.5
Br4—U1—Br3	89.792 (14)	C15—C16—H16B	109.5
Br5—U1—Br3	175.601 (13)	H16A—C16—H16B	109.5
Br6—U1—Br3	89.760 (14)	C15—C16—H16C	109.5
Br1—U1—Br3	86.593 (13)	H16A—C16—H16C	109.5
C11—N1—C13	114.4 (4)	H16B—C16—H16C	109.5
C11—N1—C15	109.2 (3)	N2—C21—C22	113.4 (4)
C13—N1—C15	113.5 (3)	N2—C21—H21A	108.9
C11—N1—H1	106.3	C22—C21—H21A	108.9
C13—N1—H1	106.3	N2—C21—H21B	108.9
C15—N1—H1	106.3	C22—C21—H21B	108.9
C25—N2—C21	110.5 (3)	H21A—C21—H21B	107.7
C25—N2—C23	113.0 (3)	C21—C22—H22A	109.5
C21—N2—C23	113.6 (3)	C21—C22—H22B	109.5
C25—N2—H2	106.3	H22A—C22—H22B	109.5
C21—N2—H2	106.3	C21—C22—H22C	109.5
C23—N2—H2	106.3	H22A—C22—H22C	109.5
Cl2—C1—Cl1	112.5 (3)	H22B—C22—H22C	109.5
Cl2—C1—H1A	109.1	C24—C23—N2	113.3 (4)
Cl1—C1—H1A	109.1	C24—C23—H23A	108.9
Cl2—C1—H1B	109.1	N2—C23—H23A	108.9
Cl1—C1—H1B	109.1	C24—C23—H23B	108.9
H1A—C1—H1B	107.8	N2—C23—H23B	108.9
N1—C11—C12	112.8 (4)	H23A—C23—H23B	107.7
N1—C11—H11A	109.0	C23—C24—H24A	109.5
C12—C11—H11A	109.0	C23—C24—H24B	109.5
N1—C11—H11B	109.0	H24A—C24—H24B	109.5
C12—C11—H11B	109.0	C23—C24—H24C	109.5
H11A—C11—H11B	107.8	H24A—C24—H24C	109.5
C11—C12—H12A	109.5	H24B—C24—H24C	109.5
C11—C12—H12B	109.5	C26—C25—N2	112.8 (4)
H12A—C12—H12B	109.5	C26—C25—H25A	109.0
C11—C12—H12C	109.5	N2—C25—H25A	109.0
H12A—C12—H12C	109.5	C26—C25—H25B	109.0
H12B—C12—H12C	109.5	N2—C25—H25B	109.0
C14—C13—N1	113.4 (4)	H25A—C25—H25B	107.8
C14—C13—H13A	108.9	C25—C26—H26A	109.5
N1—C13—H13A	108.9	C25—C26—H26B	109.5
C14—C13—H13B	108.9	H26A—C26—H26B	109.5
N1—C13—H13B	108.9	C25—C26—H26C	109.5
H13A—C13—H13B	107.7	H26A—C26—H26C	109.5
C13—C14—H14A	109.5	H26B—C26—H26C	109.5
C13—C14—H14B	109.5		
			
C13—N1—C11—C12	−54.2 (5)	C25—N2—C21—C22	−168.9 (4)
C15—N1—C11—C12	177.2 (4)	C23—N2—C21—C22	62.8 (5)
C11—N1—C13—C14	−61.4 (5)	C25—N2—C23—C24	−56.5 (5)
C15—N1—C13—C14	64.9 (5)	C21—N2—C23—C24	70.5 (5)
C11—N1—C15—C16	−171.9 (4)	C21—N2—C25—C26	171.9 (4)
C13—N1—C15—C16	59.1 (5)	C23—N2—C25—C26	−59.4 (5)

### Hydrogen-bond geometry (Å, º)

**Table:**

*D*—H···*A*	*D*—H	H···*A*	*D*···*A*	*D*—H···*A*
N1—H1···Br3	1.00	2.71	3.480 (3)	134
N1—H1···Br5^i^	1.00	2.83	3.658 (3)	141
C11—H11*A*···Br2	0.99	3.13	4.070 (5)	158
C11—H11*B*···Br4^ii^	0.99	3.24	4.184 (4)	160
C13—H13*B*···Br6^i^	0.99	3.38	4.339 (5)	163
C14—H14*A*···Br2^ii^	0.98	3.38	4.350 (6)	173
C14—H14*B*···Cl1^i^	0.98	2.95	3.922 (5)	169
C16—H16*B*···Cl2^iii^	0.98	2.97	3.904 (5)	160
N2—H2···Br1	1.00	2.59	3.499 (4)	152
C21—H21*B*···Br5^i^	0.99	3.12	4.005 (5)	150
C22—H22*C*···Br4	0.98	3.20	4.139 (5)	162
C23—H23*A*···Cl1	0.99	2.88	3.865 (5)	171
C25—H25*B*···Br5^i^	0.99	3.00	3.886 (4)	149
C1—H1*B*···Br3^iv^	0.99	2.81	3.729 (5)	155

Symmetry codes: (i) −*x*+3/2, *y*−1/2, −*z*+1/2; (ii) −*x*+1, −*y*+1, −*z*; (iii) −*x*+1, −*y*+1, −*z*+1; (iv) −*x*+2, −*y*+1, −*z*+1.
